# Effect of the Degree of Li_3_PO_4_ Vapor Dissociation on the Ionic Conductivity of LiPON Thin Films

**DOI:** 10.3390/membranes13100847

**Published:** 2023-10-23

**Authors:** Alexander Kamenetskikh, Nikolay Gavrilov, Alexey Ershov, Petr Tretnikov

**Affiliations:** Institute of Electrophysics of the Ural Branch of the Russian Academy of Science, 620016 Ekaterinburg, Russia; gavrilov@iep.uran.ru (N.G.); aleksejerschov@yandex.ru (A.E.); tpetr@iep.uran.ru (P.T.)

**Keywords:** solid electrolyte, LiPON, anodic evaporation, ionic conductivity

## Abstract

Thin films of solid-state lithium-ion electrolytes show promise for use in small-sized autonomous power sources for micro- and nanoelectronic elements. The high rate of vacuum-plasma synthesis (~0.5 μm/h) of lithium phosphor-oxynitride (LiPON) films with an ionic conductivity of ~2·10^−6^ S/cm is achieved through anodic evaporation of Li_3_PO_4_ in a low-pressure arc. The microstructure and ionic conductivity of LiPON films are influenced by the proportion of free lithium in the vapor flow. This paper presents the results of a study on the plasma composition during anodic evaporation of Li_3_PO_4_ in a discharge with a self-heating hollow cathode and a crucible anode. A method is proposed for adjusting the free lithium concentration in the gas-vapor (Li_3_PO_4_ + N_2_/Ar) discharge plasma based on changing the frequency of collisions of electrons with Li_3_PO_4_ vapor in the anodic region of the discharge. It is demonstrated that an increase in the proportion of free lithium in the flow of deposited particles leads to an enhancement in the concentration and mobility of lithium ions in the deposited films and, subsequently, an improvement in the ionic conductivity of LiPON films.

## 1. Introduction

In recent years, there has been active research aimed at creating and improving power supplies for wireless microelectronic devices. One potential candidate for the role of micro-energy accumulators is planar all-solid-state lithium-ion micro-batteries [1]. Such batteries consist of a multilayer structure formed by thin films of the anode, cathode, and solid electrolyte. One promising solid electrolyte for these batteries is lithium phosphorus-oxynitride (LiPON). It offers several key advantages, including electrochemical stability over a wide potential range of 0–5 V, thermal stability at temperatures up to 300 °C, and compatibility of the LiPON film deposition process with semiconductor and MEMS technologies [2].

The primary method for depositing LiPON films is high-frequency magnetron sputtering of Li_3_PO_4_ in an N_2_ medium. The initial ionic conductivity of the films, as reported in [3], was ~3.3 × 10^−6^ S/cm at room temperature. Numerous studies have explored the influence of various synthesis conditions (nitrogen pressure, sample temperature, ion energy bombarding, and growing film) on the ionic conductivity of LiPON, but these efforts have not yielded consistent results [4]. A theoretical study using molecular dynamics [5] helped resolve some of the contradictions in the experimental data. The key findings of this study [5] can be summarized as follows: an increase in ionic conductivity is achieved by enhancing the mobility of Li^+^ ions through (1) the formation of bonds between structural elements containing embedded nitrogen (Li(O,N)_4_ and P(O,N)_4_), (2) the substitution of O with less electronegative N, which weakens the interaction with Li^+^, and (3) the introduction of an excessive amount of Li. While a substantial body of experimental data exists on the effect of embedded nitrogen on the properties of LiPON (for example, [6,7,8,9,10,11]), research on producing films with an excessive concentration of Li remains limited.

To increase the concentration of Li in LiPON films, the sputtered target Li_3_PO_4_ is doped with Li_2_O [12,13]. An increase in the ionic conductivity of films up to 6.4 × 10^−6^ S/cm was observed with an increase in the molar ratio of Li_2_O:Li_3_PO_4_ up to 2:1 [13]. However, with a larger amount of Li_2_O, the ionic conductivity decreased to 2.1 × 10^−6^ S/cm, and Li segregation was observed, resulting in the formation of spherical inclusions around which the film was destroyed. In [14], films with an increased Li/P ratio (~3.8) were deposited outside the active region of a magnetron discharge. Despite the extremely low values of the N/P ratio (less than 0.1), the ionic conductivity of the films reached 6.7 × 10^−6^ S/cm, which was explained by the formation of a structure characterized by increased ion mobility. It is important to note that all the experimental data provided pertained to films obtained by RF magnetron sputtering with a low deposition rate (~ 50 nm/h for LiPON).

This study describes the modernization of the device for LiPON film deposition using the method of reactive anodic evaporation of Li_3_PO_4_ in a low-pressure arc [15]. The utilization of separate plasma sources in the modified electrode system for heating the crucible and vapor dissociation enabled the controlled alteration of the concentration of free Li in the flow of vaporized particles. This allowed for a comprehensive investigation of the vapor-gas-mixture generation and Li_3_PO_4_ vapor decomposition in the arc plasma. The dissociation of Li_3_PO_4_ molecules in plasma results in changes in the elemental composition of films, their morphology, and ionic conductivity.

## 2. Materials and Methods

The method used for depositing LiPON solid-electrolyte films is based on the thermal evaporation of Li_3_PO_4_ and vapor deposition onto a substrate in Ar/N_2_ plasma of a low-pressure arc. The high-intensity evaporation allows for significantly higher film deposition rates compared with the magnetron-sputtering method [15]. The method enables independent control of the evaporator temperature and, consequently, the vapor pressure of Li_3_PO_4_, as well as the frequency of collisions of electrons with vapor in the arc discharge plasma, which affects the concentration of free lithium in the plasma. The interaction of vaporized Li_3_PO_4_ molecules in the discharge gap of the arc with the discharge plasma electrons leads to the dissociation of the molecules and the release of free Li in the particle flow deposited on the substrate.

**Figure 1:**
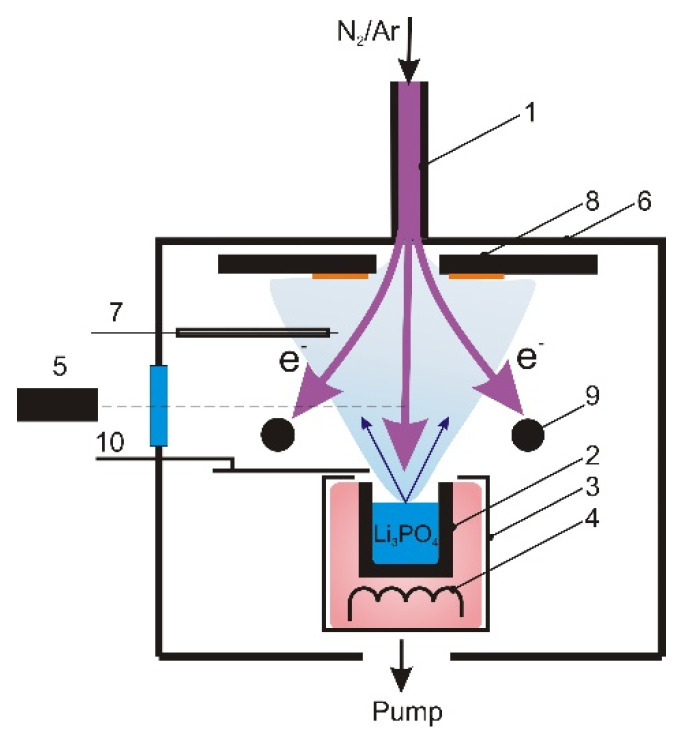
Scheme of the experimental installation: 1—self-heated hollow cathode; 2—anodic crucible; 3—thermionic cathode; 4—screen; 5—spectrometer; 6—vacuum chamber; 7—Langmuir probe; 8—sample holder; 9—additional anode; 10—shutter.

The experimental setup is shown in Figure 1. LiPON films were deposited in a gas-discharge system consisting of a self-heating hollow cathode (SHHC) 1 and a crucible anode 2. A combination of two methods was used to heat the anodic crucible to temperatures at which the Li_3_PO_4_ powder placed inside the crucible (mass fraction 99.5%) would evaporate: heating in the main discharge with an SHHC and heating in the auxiliary discharge with a thermionic cathode 3. The plasma of the auxiliary discharge was confined within a screen 4, preventing its interaction with the Li_3_PO_4_ vapors. The dissociation of vapors was facilitated by the flow of electrons from the discharge plasma with the SHHC. The current of the main discharge (ranging from 0–3 A) and the power of the auxiliary discharge could be independently regulated, enabling adjustments to the frequency of the electron–vapor collisions while maintaining a constant heating power and vapor pressure. The temperature of the anodic crucible was monitored using a chromel–alumel thermocouple.

The discharge plasma was analyzed through optical emission spectroscopy and probe diagnostics. The HR4000 high-resolution spectrometer (OceanOptics) 5 with a spectral range of 200–1100 nm was positioned on the side surface of the vacuum chamber 6, with a distance of 350 mm from the axis of the gas discharge system to the input aperture of the device. Plasma potential and electronic temperature were measured using a Langmuir collecting probe 7 made of W-wire with a diameter of 0.6 mm. The probe was situated within a ceramic screen to prevent direct vapor deposition. Before each measurement, the probe surface was cleaned by heating it to high temperatures. During one measurement cycle (lasting no more than 1 s), 20 probe characteristics were recorded, and plasma parameters were determined as average values.

LiPON films were deposited at a pressure of a mixture of Ar/N_2_ gases equal to 0.3 Pa, with the partial pressure of N_2_ at 0.2 Pa. Gas was supplied to the working camber through the SHHC. The films were deposited on polished substrates with a diameter of 20 mm made of 12Cr18Ni10Ti steel. These substrates were placed on a holder 8 mounted at a distance of 12 cm from the anodic crucible.

Before film deposition, the substrates underwent cleaning in an ultrasonic bath using an acetone solution and were subsequently dried in an N_2_ flow. The vacuum chamber was evacuated to a pressure of 1 × 10^−3^ Pa using a turbomolecular pump with a pumping rate of 500 L/s. Ion sputtering of the substrates was performed for 10 min in an Ar plasma of a discharge maintained between the SHHC and an additional anode 9. The current density and ion energy were 3 mA/cm^2^ and ~500 eV, respectively. The crucible was heated to operating temperatures within a discharge that featured a thermionic cathode with a closed shutter 10. The films were deposited at a rate of ~0.5 µm/h by evaporation of Li_3_PO_4_ from the solid phase.

After the film deposition, the samples were transferred to an installation equipped with a magnetron-sputtering system, and a 12Cr18Ni10Ti contact layer was deposited. The ionic conductivity of LiPON films in the symmetric capacitor structures obtained was measured using electrochemical impedance spectroscopy [16] employing the potentiostat P-45X (Chernogolovka, Russia). The signal amplitude was 0.15 V, and the frequency range spanned 0.3–1 × 10^6^ Hz. The characteristics obtained in the coordinates of imaginary and real impedance (Nyquist diagrams) included a linear section in the low-frequency region due to the double layer at the “electrolyte–electrode” interface and a semicircle in the high-frequency region representing the impedance of the film. This was used to determine the electrical resistance of the film (R_el_). The ionic conductivity of the films (σ_i_) was calculated as the ratio σ_i_ = d/R_el_ A, where d represents the film thickness and A denotes the contact area.

## 3. Results

**Figure 2:**
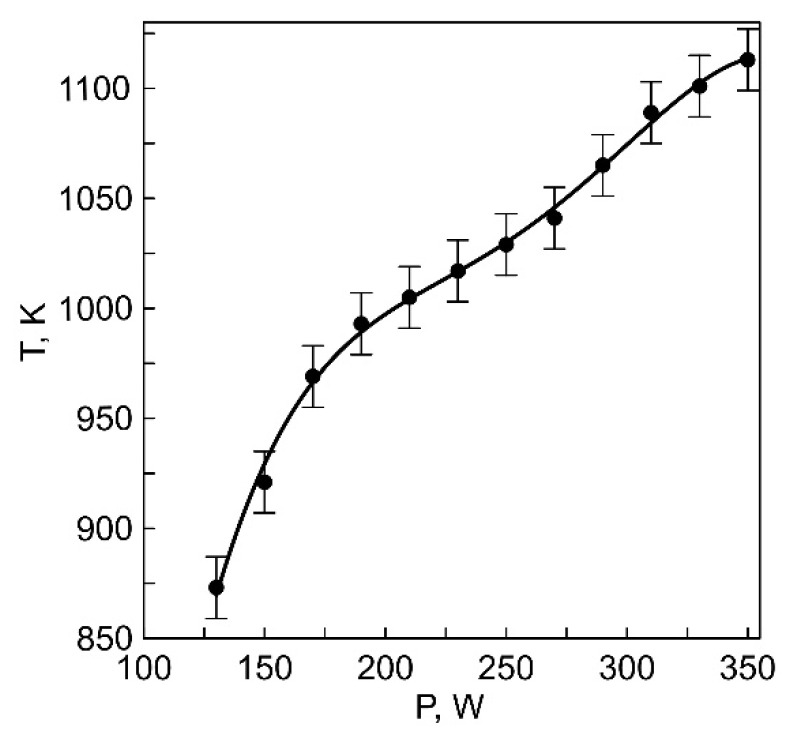
Dependence of the temperature of the anodic crucible on the discharge power with a thermionic cathode.

The relationship between the temperature of the anodic crucible (T), heated in an auxiliary discharge with a thermionic cathode, and the heating power of the crucible is shown in Figure 2. At the maximum heating power (350 W), the crucible temperature reached 1100 K, which is lower than the melting point of Li_3_PO_4_ (~1500 K [17]). Since the Li_3_PO_4_ powder is heated indirectly, it can be inferred that evaporation occurs from the solid phase. During the transition to the liquid state, the melt assumes a spherical shape because Li_3_PO_4_ does not wet graphite.

**Figure 3:**
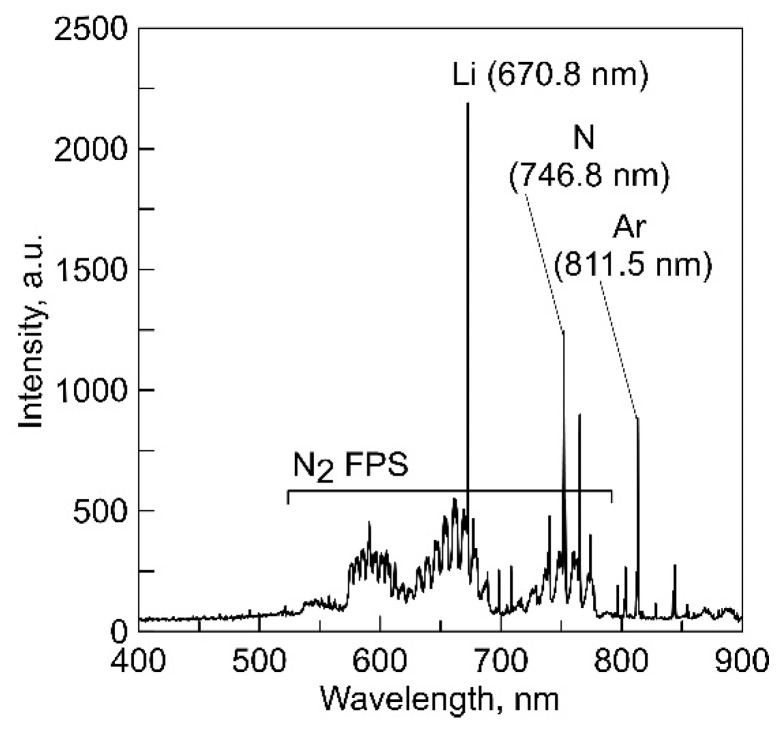
Overview spectrum of optical plasma emission.

The relationship between vapor pressure and the crucible temperature was determined using optical emission spectroscopy. The crucible was heated in an auxiliary discharge, while the main discharge with a current of 5 A was maintained between the SHHC 1 and the additional anode 9 (see Figure 1). The overview spectrum of optical plasma emission is presented in Figure 3.

**Figure 4:**
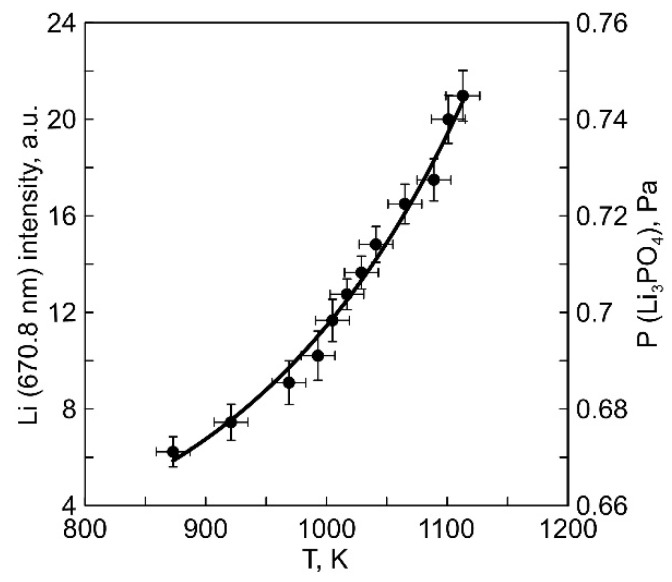
Dependence of the intensity of the optical emission line Li (670.8 nm) on the temperature of the anodic crucible.

The most intense Li line (670.8 nm) in the spectrum corresponds to the transition of the Li atom to the ground state ^2^p^o^- > ^2^S_1/2_ with an upper-level energy of 1.84 eV. As the temperature of the anodic crucible increases proportionally to the discharge power between the thermionic cathode 3 and the anodic crucible 2, there is an increase in the intensity of this line, as illustrated in (Figure 4). The line intensity was determined as the average of five measurements performed automatically during an exposure time of ~1500 ms. The resulting relationship was calibrated numerically (Appendix A) and plotted on a graph with the vapor pressure of Li_3_PO_4_ and the temperature of the anodic crucible as coordinates. The estimated evaporation rate of Li_3_PO_4_, calculated based on the reduction in the mass of material in the crucible, was 1.4 × 10^−2^ g/(m^2^·s) at a discharge power of 350 W. The range of calculated vapor pressure values for the crucible temperature (850–1100 K) was 0.66–0.74 Pa.

**Figure 5:**
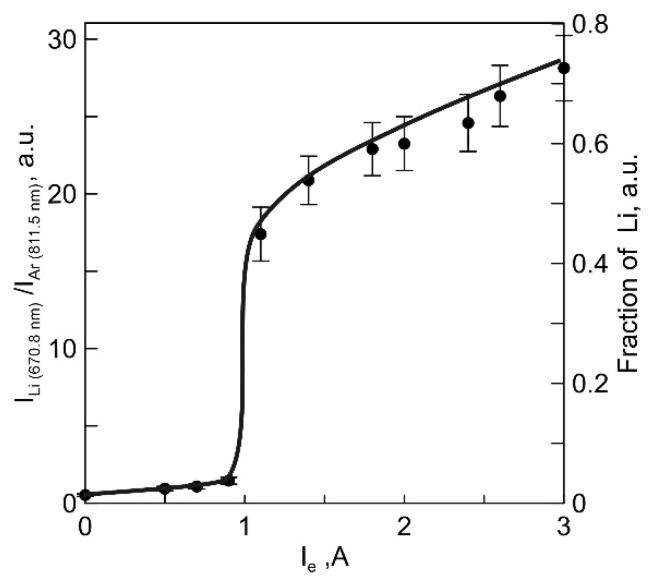
Dependence of the ratio of the intensities of the Li (670.8 nm) and Ar (811.5 nm) lines (the fraction of free Li) on the discharge current between the SHHC and the anodic crucible.

When a discharge current is switched to the anodic crucible through an area with an elevated vapor pressure, the extent of decomposition of Li_3_PO_4_ vapors increases. In the study examining the impact of the main discharge current on vapor dissociation, the main discharge current to the anodic crucible was adjusted within the range of 0–3 A, and any concurrent increase in the heating power of the anodic crucible was counterbalanced by reducing the discharge power with a thermionic cathode to maintain a consistent temperature of the anodic crucible. The method of optical actinometry [18] was employed to estimate the concentration of free Li in the vapor dissociated (Appendix B). The relationship between the intensity ratio of the Li (670.8 nm) and Ar (811.5 nm) lines and the discharge current between the SHHC and the anodic crucible is illustrated in Figure 5.

The data obtained reveals that the concentration of free Li in the plasma of the vapor-gas mixture consistently rises with an increase in electron current. The most significant increase in the dissociation of Li_3_PO_4_ vapors occurs at a current value of approximately ~1 A. The proportion of free Li in the vapor was estimated as the ratio of Li concentration to the concentration of Li_3_PO_4_ vapor determined from experimental data (as described in Appendix B). In the absence of electron flow to the anodic crucible from the discharge plasma with the SHHC, the proportion of free Li in the vapors was ~1.6%. As the electron current increased to 3 A, the proportion of free Li increased to ~65%.

**Figure 6:**
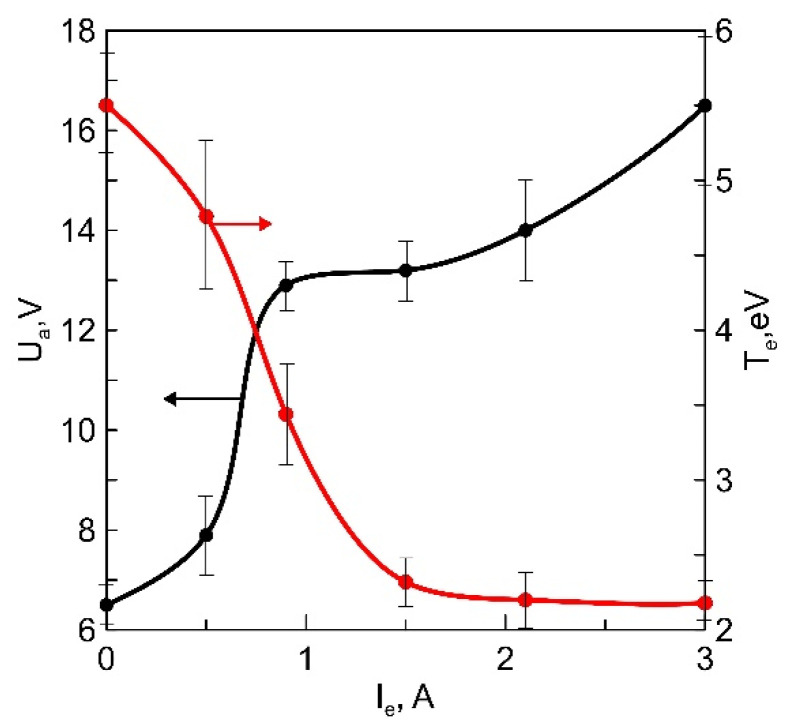
Dependencies of the positive anodic potential drop and the electron temperature of the plasma on the discharge current between the SHHC and the anodic crucible.

The results of probe measurements presented in Figure 6 indicate a sharp increase in the positive anodic potential drop at the crucible within the current range of 0–1 A and a reduction in the temperature of plasma electrons. It can be assumed that the increase in the positive anodic drop at the crucible results in a higher frequency of dissociation of vapor molecules. Consequently, the resulting increase in the concentration of free Li in the plasma leads to a reduction in the electron temperature of the vapor-gas-mixture plasma.

**Figure 7:**
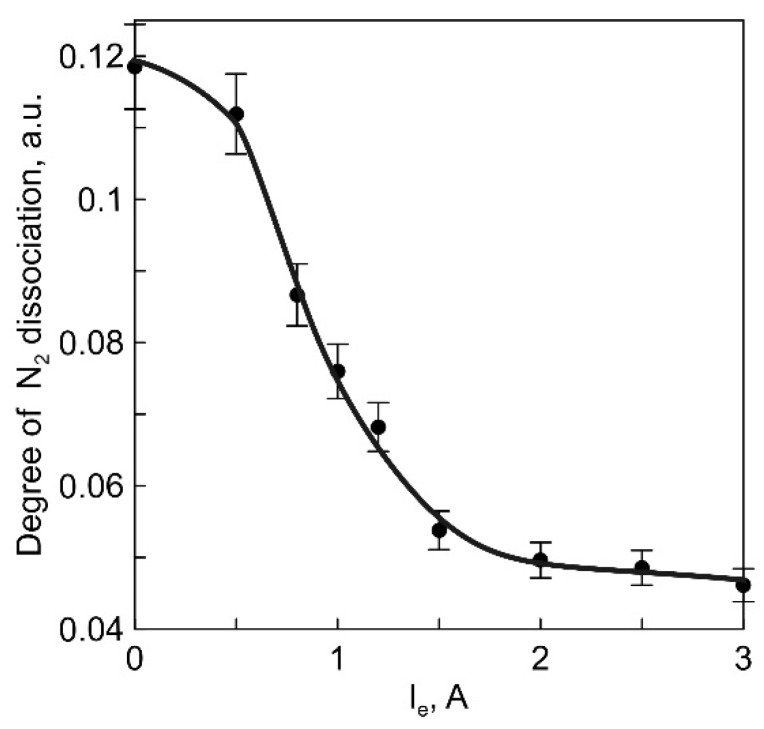
Dependence of the degree of dissociation of N_2_ on the discharge current between the SHHC and the anodic crucible.

To determine the change in the concentration of atomic nitrogen, the intensity of the N (746.8 nm) line in the optical emission spectra of plasma was measured. The degree of dissociation of N_2_ was determined using optical actinometry, where Ar (811.5 nm) was used as an actinometer [19]. As the discharge current between the SHHC and the anodic crucible increased to 3 A, the degree of dissociation of N_2_ decreased from 12% to 4% (Figure 7).

This decrease can be attributed to a reduction in the electron temperature of the plasma (as shown in Figure 6) and a more rapid decrease in the rate of the dissociation reaction of N_2_ compared with the increase in the current [20].

**Figure 8:**
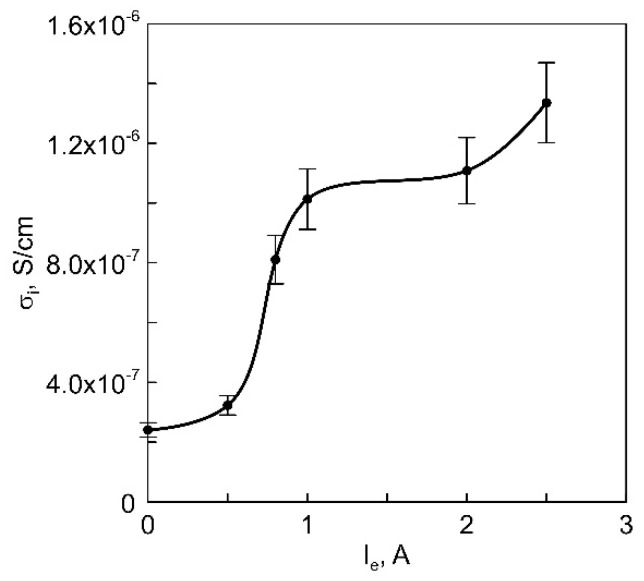
The dependence of the ionic conductivity of LiPON films on the discharge current between the SHHC and the anodic crucible.

The relationship between the ionic conductivity of LiPON films and the main discharge current to the anodic crucible is shown in Figure 8. As the proportion of free Li in the vapor-gas plasma increases, the ionic conductivity of the films rises from 2.4 × 10^−7^ to 1.3 × 10^−6^ S/cm.

## 4. Discussion

Thin films made of nitrogen-doped Li_3_PO_4_ were first studied in [3]. In that study, an increase in the N/P ratio in films from 0 to 0.46 was accompanied by an increase in ionic conductivity from 7 × 10^−8^ to 3.3 × 10^−6^ S/cm. The development of ideas about the mechanism of increasing ionic conductivity by doping Li_3_PO_4_ with nitrogen led to a model in which introduced nitrogen destabilizes the positions of Li in the LiPON structure, increases the energy of particles, and thereby reduces the activation energy of diffusion of Li^+^ ions [5]. This model’s validity is supported by the results of a comprehensive study of the transport properties of LiPON films performed by nuclear magnetic resonance [21], which demonstrates that an increase in the concentration of N in the films boosts the mobility of Li^+^ and creates conditions for an increase in ionic conductivity.

The results of studies of LiPON films with an extremely low N/P ratio < 0.1 indicate the decisive effect of Li^+^ mobility [14]. When films were formed outside the active region of the magnetron discharge, the concentration of N significantly decreased, while the ionic conductivity increased to ~6.7 × 10^−6^ S/cm. Despite the excess Li/P ratio of ~3.8 in the films, the proportion of free Li+ ions contributing to ionic conductivity was close (0.053%) to that for samples obtained through the standard film deposition procedure using RF magnetron sputtering (0.067%). However, the mobility of Li^+^ in such films was four times higher (1.2 × 10^−6^ cm^2^/(V·s)). The results of [14] suggest that the LiPON structure, characterized by enhanced ion mobility and ionic conductivity, is formed with an increase in the concentration of Li, not solely through N doping. This conclusion is corroborated by numerous experiments using sputtering targets enriched with Li, such as those created by adding Li_2_O to the Li_3_PO_4_ composition of the target [12,13].

In this paper, a method of increasing the concentration of Li in films is used, which differs fundamentally from the methods used in magnetron systems and the above-mentioned method involving the addition of Li_2_O to the target. The change in the concentration of free Li occurs in the plasma as a result of the decomposition of a portion of the vapor molecules. The Li_3_PO_4_ vapor, with a pressure reaching 0.74 Pa, is affected by the flow of electrons from the discharge plasma with the SHHC, a significant proportion of which has enough energy to break the Li bonds in Li_3_PO_4_ through direct electron impact [22,23]. The frequency of collisions leading to dissociation is proportional to the discharge current, the concentration of vapor particles, and the cross-section of the process (σ(E)), which is a non-monotonic function of the electron energy (E). An increase in the positive anodic potential drop near the anodic crucible increases E and σ(E). A sharp increase in the number of free Li atoms at a constant vapor concentration is observed with an increase in the anodic drop from 8 to 13 V (Figure 6) as a result of a significant increase in σ(E) in this energy range. In the spectra of plasma optical emission from the vapor-gas mixture (Figure 3), oxygen and phosphorus lines are not detected, indicating the predominant cleavage of Li atoms. The increase in the proportion of Li in the particle flow can be attributed to differences in the nature of particle movement toward the substrate. As the gas-kinetic cross-section of Li atoms is an order of magnitude smaller than the cross-section of the remaining particles in the vapor-gas mixture, this ensures their movement in a direct flight mode, while the concentration of other particles in the flow decreases due to multiple scattering as they move toward the surface.

An increase in the proportion of Li in the particle flow is accompanied by an increase in the ionic conductivity of LiPON films. Numerical calculations of the concentration (n_i_) and mobility of charges (μ_i_) were performed using the model [14] (see Appendix C). From the calculation results shown in Table 1, it is evident that an increase in the proportion of free Li in the particle flow leads to an increase in both the concentration ni of Li^+^ ions in films and their mobility μ_i_. The values of n_i_ and μ_i_ are of the same order of magnitude as those obtained in [14]. However, unlike films deposited by RF magnetron sputtering, which differ only in the value of μ_i_, for LiPON films deposited under conditions of Li_3_PO_4_ vapor decomposition by electron flux, increases in n_i_ and μ_i_ were observed in approximately equal proportions.

It is worth noting that the degree of N_2_ dissociation also plays a role in influencing the ionic conductivity of LiPON. It has been observed that an increased concentration of atomic nitrogen contributes to an increase in the ionic conductivity of LiPON films deposited through RF magnetron sputtering [9]. In the experiments described earlier, along with an increase in the proportion of free Li in the vapor-gas plasma, a decrease in the degree of N_2_ dissociation at a constant gas pressure is observed. This change is a result of the reduced electron temperature of the plasma and the corresponding decrease in the rates of reactions leading to the formation of atomic nitrogen [20]. Therefore, the primary factor leading to an increase in the ionic conductivity of LiPON films deposited under conditions of Li_3_PO_4_ vapor decomposition by electron flow is the increase in the proportion of free Li in the flow of deposited particles, which, in turn, results in a simultaneous increase in the mobility and concentration of Li^+^ within the films.

## 5. Conclusions

The primary impact of Li_3_PO_4_ vapor dissociation in plasma on the performance of LiPON films deposited by the anodic evaporation of Li_3_PO_4_ in a discharge with a self-heated hollow cathode and anodic crucible has been demonstrated. The concentration of free Li in a vapor-gas (Li_3_PO_4_ + N_2_/Ar) discharge plasma was regulated by adjusting the frequency of electron collisions with Li_3_PO_4_ vapors in the anodic region of the discharge. It was found that the proportion of free Li in vapor-gas plasma reached 65% at a vapor pressure of ~0.7 Pa, anodic-crucible electron current of 3 A, and a positive anodic potential drop at the crucible of 15 V. An increase in the concentration of free lithium in the film initially leads to an increase in its ionic conductivity, and with excessively high concentrations of free lithium, segregation begins, significantly deteriorating the film quality.

At nearly the optimal degree of Li_3_PO_4_ vapor dissociation, LiPON films with ionic conductivity of up to 1.4 × 10^−6^ S/cm were synthesized. In contrast to LiPON films deposited through magnetron sputtering, where changes in ionic conductivity are primarily attributed to alterations in the mobility of Li ions, the increase in ionic conductivity of the films deposited in the vapor-gas plasma of a low-pressure arc is due to a simultaneous increase in the concentration and mobility of Li ions.

## Figures and Tables

**Table 1:** Calculation results.

The Discharge Current between SHHC and Anodic Crucible, A	σ_i_, 10^−6^ S/cm	n_i_, 10^−19^ cm^−3^	μ_i_, 10^7^ cm^2^/(V·s)
0	0.24	0.4	3.1
0.5	0.32	0.48	5.1
0.8	0.81	0.85	5.9
1	1.01	1.02	6.2
2	1.1	4.5	9.3
2.5	1.4	7.9	13.2

## Data Availability

Not applicable.

## Appendix A

The method for calculating the Li_3_PO_4_ vapor pressure generated during anodic evaporation in a low-pressure arc is described below.

Assuming that the frequency of dissociation of vapor molecules is proportional to the main discharge current, which was kept constant in the experiment, and the vapor density, the dependence presented in Figure 4 may be interpreted as a function of vapor pressure (P) from the temperature of the crucible (T) in accordance with logarithmic law [24]:

lgP = A + B·T + C/T + D/T^2^ + E·lgT, (A1)

where A, B, C, D, and E are constants.

Calibration of the pressure scale was carried out by the calculation method.

The relationship of the evaporation rate with the saturated vapor pressure is determined by the Hertz–Knudsen equation [25]:

dN/dt = SP (2πmkT)^−1/2^, (A2)

where N is the number of atoms leaving the surface area S; m is the mass of these atoms; and k is the Boltzmann constant. The evaporation rate of Li_3_PO_4_, estimated by the decrease in the mass of the material in the crucible, was 1.4 × 10^−2^ g/(m^2^·s) at a discharge power of 350 W. The values of the constants A, B, C, D, and E, determined using the calculated values of vapor pressure, were A = −1.65; B = −4.08 × 10^−9^; C = 0.01; D = −1.97; and E = 0.5, respectively.

## Appendix B

The method of optical actinometry [18] was used to estimate the change in the concentration of free Li in a wide range of values of the main discharge current, which makes it possible to identify the influence of additional factors on the concentration of excited particles, excluding the influence of changes in plasma density with an increase in the discharge current. The method considers the change in the intensity of the line of the studied medium component (I_Li_) relative to the intensity (I_Ar_) of the component, the concentration (n_Ar_) of which is a known and set constant. The concentration values of the studied component (n_Li_) are determined from the ratio

n_Li_/n_Ar_ = K I_Li_/I_Ar_, (A3)

where the coefficient K does not depend on the discharge parameters and is determined by the parameters of the optical transitions and the thermal energy of the excited particles.

The Ar line (811.5 nm) was used in the measurements corresponding to a state with an excitation cross-section similar to Li [26,27]. The value of K was determined using known data from the database [28]. The proportion of free Li in the vapor was estimated as the ratio of n_Li_ and the concentration of Li_3_PO_4_ vapor determined from experimental data (Appendix A).

## Appendix C

The method to estimate charge-carrier concentration (n_i_) and mobility (μ_i_) is described below.

Nyquist diagrams of LiPON films deposited at different electron currents of the main discharge were analyzed in ZView [29] using an equivalent circuit including contact resistance; constant phase elements characterizing the electrical capacitance of the C_el_ film and the C_dl_ double layer that occurs at the boundary of the film and substrate (interface); and the electrical resistance of the electrolyte and the interface area. The relative permittivity of the electrolyte (ε) and the Debye length (L_D_) were determined as

ε = C_el_/ε_0_ × d/A, (A4)

L_D_ = d × (C_el_/C_dl_), (A5)

where d is the LiPON film thickness and A is the electrical contact area. The values of n_i_ and μ_i_ were determined from the equations

n_i_ = ε × ε_0_ × kT_m_/(L_D_^2^ × e^2^), (Tm = 300 K); (A6)

μ_i_ = σ_i_/(n_i_ × e), (A7)

where σ_i_ is the ionic conductivity of the film.
